# How do physicians decide to treat: an empirical evaluation of the threshold model

**DOI:** 10.1186/1472-6947-14-47

**Published:** 2014-06-05

**Authors:** Benjamin Djulbegovic, Shira Elqayam, Tea Reljic, Iztok Hozo, Branko Miladinovic, Athanasios Tsalatsanis, Ambuj Kumar, Jason Beckstead, Stephanie Taylor, Janice Cannon-Bowers

**Affiliations:** 1 Department of Internal Medicine, Division of Evidence-based Medicine and Health Outcomes Research, University of South Florida, Tampa, FL, USA; 2 Department of Health Outcomes and Behavior, Moffitt Cancer Center & Research Institute, Tampa, FL, USA; 3 Department of Hematology, Moffitt Cancer Center & Research Institute, Tampa, FL, USA; 4 De Montfort University, Leicester, UK; 5 Indiana University Northwest, Department of Mathematics, Gary, IN, USA; 6 College of Nursing, University of South Florida, Tampa, FL, USA; 7 Center for Advanced Medical Learning & Simulations, University of South Florida, Tampa, FL, USA; 8 USF Health, 3515 East Fletcher Avenue, MDT 1202, Tampa, FL 33612, USA

**Keywords:** Medical decision-making, Threshold model, Dual-processing theory, Regret, Expected utility theory

## Abstract

**Background:**

According to the threshold model, when faced with a decision under diagnostic uncertainty, physicians should administer treatment if the probability of disease is above a specified threshold and withhold treatment otherwise. The objectives of the present study are to a) evaluate if physicians act according to a threshold model, b) examine which of the existing threshold models [expected utility theory model (EUT), regret-based threshold model, or dual-processing theory] explains the physicians’ decision-making best.

**Methods:**

A survey employing realistic clinical treatment vignettes for patients with pulmonary embolism and acute myeloid leukemia was administered to forty-one practicing physicians across different medical specialties. Participants were randomly assigned to the order of presentation of the case vignettes and re-randomized to the order of “high” versus “low” threshold case. The main outcome measure was the proportion of physicians who would or would not prescribe treatment in relation to perceived changes in threshold probability.

**Results:**

Fewer physicians choose to treat as the benefit/harms ratio decreased (i.e. the threshold increased) and more physicians administered treatment as the benefit/harms ratio increased (and the threshold decreased). When compared to the actual treatment recommendations, we found that the regret model was marginally superior to the EUT model [Odds ratio (OR) = 1.49; 95% confidence interval (CI) 1.00 to 2.23; p = 0.056]. The dual-processing model was statistically significantly superior to both EUT model [OR = 1.75, 95% CI 1.67 to 4.08; p < 0.001] and regret model [OR = 2.61, 95% CI 1.11 to 2.77; p = 0.018].

**Conclusions:**

We provide the first empirical evidence that physicians’ decision-making can be explained by the threshold model. Of the threshold models tested, the dual-processing theory of decision-making provides the best explanation for the observed empirical results.

## Background

Medical decision-making is often performed under conditions of diagnostic uncertainty; that is, physicians frequently need to decide whether to give treatment to a patient who may or may not have a disease. Clinical practice is full of these examples. For instance, if the physician treating a patient with a sore throat estimates that the probability of streptococcal infection is sufficiently high, she may decide to treat – assuming that the benefits of administering antibiotic outweigh its potential harms. Thus, to make appropriate therapeutic decision when a diagnosis is uncertain, the clinician has to: 1) ascertain the probability of a patient having the disease, and 2) decide whether the potential treatment benefits will outweigh its harms.

In everyday clinical practice, the assessment of the likelihood of disease and balance of treatment’s benefits and harms is often done intuitively, but this decision-making process can be formalized under the “threshold model” [1,2]. According to the threshold model, when faced with uncertainty about whether to treat a patient who may or may not have a disease, there must exist some probability at which a physician is indifferent between administering versus not administering treatment; this is known as threshold probability [1,2]. Physicians would choose to treat when the probability of disease is above the threshold probability and would choose to withhold treatment otherwise [1,2]. The threshold model stipulates that as the therapeutic benefit/harms ratio increases, the threshold probability at which treatment is justified is lowered. Conversely, if the treatment’s benefit/harms ratio decreases, the required threshold for therapeutic action will be higher. To date, three types of threshold models have been described: 1) the original model, based on the expected utility theory (EUT) framework (T_EUT_) [1,2]; 2) the regret-based threshold model (T_RG_) [3-5]; and 3) the threshold model based on the dual-processing theory of decision-making (T_DP_) [6].

The T_EUT_ model is derived from the principles of decision theory, which hold that a decision-maker should select the option with the highest expected utility to maximize achievement of valued outcomes. The T_RG_ model is based on expected regret theory, which holds that the preferred course of action is based on the least amount of regret associated with a possibly wrong decision. The T_DP_ model is based on dual processing theories, which postulate that our cognition is governed by so called type 1 or 2 processes [7-15]. Type 1 processes are intuitive, automatic, fast, narrative, experiential and affect-based; type 2 processes are analytical, slow, verbal, and deliberative supporting formal logical and probabilistic analyses [7-16].

Despite the widespread popularity, none of the threshold models (T_EUT,_ T_RG,_ T_DP_) have been submitted to empirical evaluation to test their descriptive accuracy. The purpose of our study was to assess whether physicians act according to a threshold model, and if they do, to determine which model best explains their decision-making. Knowing if physicians operate under a threshold model and which model best describes physicians’ decisions is very important for medical education as it can help identify the most salient features of medical decision-making. This, in turn can be used for didactic purposes towards better practice of clinical decision-making. In addition, understanding the decision-making processes can help explain patterns observed in the contemporary clinical practice such as treatment overuse and underuse.

## Methods

### Participants and setting

Physicians from the University of South Florida and Evidence-based Medicine Discussion Group were recruited for the study via email invitation to participate in a web-based survey. E-mail invitations were sent via institutional listserv followed by a weekly reminder. No incentives were offered for participation in the study. The only inclusion criteria were that participants were practicing physicians, regardless of the field of medicine, actively involved in therapeutic decision-making on a daily basis. The survey was closed after the target sample was reached. The study was approved by the USF IRB (No. Pro9047).

### Design and materials

All theories of decision-making agree that choices are functions of benefits (gains) and harms (losses). Therefore, we constructed the case vignettes to allow easy discernment of benefits and harms for serious, life-threatening outcomes. The aim was to compel our study participants to rely on the estimates of benefits and harms, in particular on the benefit/harm (B/H) ratio. To minimize “framing effect” [17], we chose presentation and wording that is commonly used in the literature and medical communication and with which most physicians are familiar.

#### Threshold models

Our case vignettes refer to a clinical situation when a decision about treatment has to be made but a physician is uncertain whether the patient has a given condition and no further diagnostic tests are available to her/him to reduce the diagnostic or prognostic uncertainty. We now provide a brief outline of all 3 models:

1) Expected utility threshold model

Although often considered gold standard of rationality, violation of decision-making by EUT is well documented in literature [5,18-21]. However, one issue is rarely directly addressed: do people violate precepts of EUT because of errors due to brain processing limitations, or because EUT does not reflect the optimal decision-making perspective of the decision-maker. For example, few people can accurately multiply 3.4578*4,678; that does not, however, mean they reject (normatively) the correct answer once they perform the calculation with help of a calculator. Most people simply correct their error and accept the answer obtained after punching the numbers into a calculator. We, therefore, asked the following question: will people behave according to EUT after they are told what they should (normatively) do? Or, will they violate the rules of EUT even after they are told what is the theoretical best course of action? For this purpose, we included a number of prescriptive statements in our case vignettes based on the EUT normative calculations.

The EUT threshold was calculated as:

(1) $$T_{\text{EUT}}=1/\left(1+\frac{B_{2}}{H_{2}}\right)$$

where benefits/harms (B_2_/H_2_) refer to the objective data obtained from the literature. Thus, if B_2_/H_2_ = 9, the probability above which we should give treatment is only 10%. [The EUT model relies on type 2 processes. Hence, we used the subscript 2 in equation 1].

2) Regret threshold model

**Figure 1:**
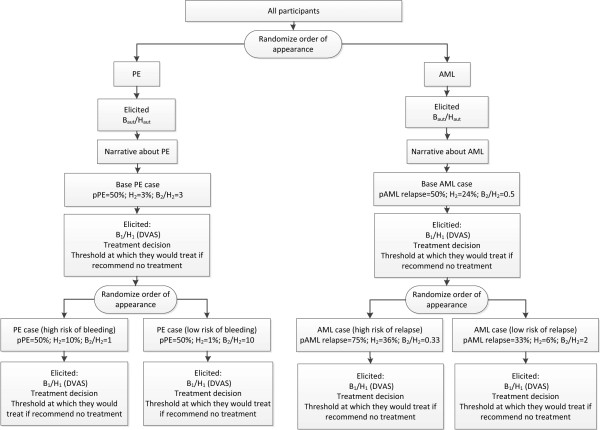
**A schema of the experimental design.** Note that design was entirely within participants and that all participants answered all question (but in different order, according to the randomization sequence). Abbreviations: PE, Pulmonary embolism; AML, Acute Myeloid Leukemia; B_aut_/H_aut_, automatic benefit to harm ratio; pPE, probability of PE, H2, harms associated with treatment provided; B_2_/H_2_, benefit to harm ratio provided in the case; B_1_/H_1_, benefit to harm ratio elicited form participants using DVAS; Dvas, dual visual analog scale; pAML, probability of AML relapse. Note: All participants completed all vignettes. Only the order of presentation of vignettes was randomized where indicated.

Many clinical decisions are driven by regret where a decision-maker (a doctor or a patient) seeks to minimize regret associated with a potentially wrong decision [3-5]. In general, in a clinical situation similar to the one considered here, a decision maker deals with two types of regret: failure to provide benefit (regret of omission) versus administering unnecessary and potentially harmful treatment (regret of commission) [3-5]. Given that in medical decision-making most decisions cannot be reversed (e.g., once surgery has occurred, its effects cannot be reversed), the T_RG_ model is based on anticipatory regret only [3-5]) (as opposed to retrospective regret or post-decision justification regret [22,23]). Anticipation of regret leads to more vigilant decision making, satisfying most of the criteria of high-quality decisions [8,24]. To estimate regret of omission versus commission, as alluded above, we employed the regret-based Dual Visual Analog Scale (DVAS) [25] (see Figure 1 and Additional file 1 for further details on actual regret elicitation). Regret threshold was calculated by employing the following formula:

(2) $$T_{\text{REG}}=1/\left(1+\frac{B_{1}}{H_{1}}\right)$$

where B_1_/H_1_ is failure to benefit/unnecessary harms. Note the regret threshold model is, psychologically, a type 1 only model, which relies on holistic assessment of benefits and harms (hence, we used subscript 1 in the equation). That is, the model predicts that the responses will be determined by regret, which is an affective (and hence type 1) response.

3) Dual-processing threshold model

In recent years, it has become evident that decision-making theories which assume a single system of reasoning are not sufficient to explain human decision-making [8,9,26-28]. Instead, as introduced above, it is increasingly accepted that cognitive processes are governed by both type 1 and type 2 processes [8,9,26-28]. We recently developed a threshold model based on dual processing theory (T_DP_), which takes into account analytical type 2 functioning based on rational calculus of EUT as well as type 1 mechanisms driven both by emotion (regret) and other type 1 processes [6].

The decision to administer treatment according to type 2 processing depends on the EUT threshold calculated as shown in equation 1. The extent of type 1 processes (i.e., the extent to which type 1 processes are not suppressed by or compete with type 2 processes) in the decision-making is given by parameter γ [0 to 1]; if γ = 0, then decision-making adheres to EUT. Conversely, if γ = 1, then type 1 processes dominate decision-making. For any 0 ≤ γ ≤ 1, decision-making is a combination of both processes. The formula for calculation of the T_DP_, is given by:

(3) $$T_{\text{DP}}=\left(T_{\text{EUT}}\right)\left[1+\frac{\gamma }{2\left(1-\gamma \right)}\left(\frac{H_{1}}{H_{2}}\right)\left(1-\frac{B_{1}}{H_{1}}\right)\right]$$

As explained, B_1_ and H_1_ are elicited from the participants (Figure 1) while T_EUT_ is calculated based on the best evidence from the literature, B_2_ and H_2_. Because γ represents the extent of activation of type 1 processes, this can be conceptualized as relative distance between analytically derived T_EUT_ and regret-based, T_REG_. Thus, we calculated γ in the following way (keeping the value between 0 and 1):

(4) $$\gamma =\left\{\begin{array}{c} \frac{T_{\text{EUT}}-T_{\text{RG}}}{T_{\text{EUT}}},\text{if}\frac{T_{\text{EUT}}-T_{\text{RG}}}{T_{\text{EUT}}}<1\\ 1,\text{otherwise} \end{array}\right)$$

Therefore, γ is equal to $\frac{T_{\text{EUT}}-T_{\text{RG}}}{T_{\text{EUT}}}$, if $\frac{T_{\text{EUT}}-T_{\text{RG}}}{T_{\text{EUT}}}<1$. If $\frac{T_{\text{EUT}}-T_{\text{RG}}}{T_{\text{EUT}}}\geq 1$, then γ is equal to 1. Estimates for γ are provided in Additional file 2, Table S1.

Note that there are many dual-processing theories [29] and the model presented here represents a specific dual-processing model that is applicable to single-point clinical decisions [6].

#### A survey to test the threshold models

We devised two clinical scenarios - one for a familiar condition and a second which required specialized knowledge. Scenario 1 was about treatment of pulmonary embolism (PE), which should be familiar to the vast majority of physicians. Scenario 2 was about treatment of acute myeloid leukemia (AML), with which only a minority of physicians have experience (see Additional file 2 for the survey/concrete examples).

To examine dual processing aspects, we used a variation of the two-response paradigm in which initial responses are considered to represent mostly type 1 processes, and later responses are considered to represent the added influence of type 2 processes. We, therefore, included more detailed information between the first and the second response.

To capture this initial (type 1) response, we first asked all participants to provide their best assessment on benefits/harms for treatment of PE and AML, respectively. That is, the first question was devoid of any case-specific contextual details. This response to benefits (B) and harms (H) due to over-learned processes (see below and Discussion) is postulated to be automatic (_aut_), and we label them here as B_aut_ and H_aut_.

The B_aut_ over H_aut_ is stipulated to serve as an “anchor” but is expected to be further modified by the contextual details of each case presentation as affected by the various type 1 and type 2 processes. By eliciting the anchor value, our attempt was to ensure elicitation of the subsequent responses related to B_1_ and H_1_ estimates within clinically realistic range. Note, however, we only need to elicit B_1_ and H_1_ values to perform the actual calculations; elicitation of B_aut_ and H_aut_ only serve to conduct the experimental procedure according to our theoretical framework.

We note that type 1 processes are determined by a number of factors, including: (a) affect, (b) evolutionary hard-wired processes, responsible for automatic responses to potential danger, (c) over-learned processes based on type 2 mechanisms that have been relegated to type 1 responses (such as the effect of intensive training resulting in the use of heuristics), and (d) the effects of tacit learning [11]. All these factors were taken into account in construction of the vignettes in the following way: medical education and exams typically consist of case vignettes, which after many hours of training become internalized and represent the basis for acquiring expertise and actual practice of medicine. The vignettes, therefore, were constructed to be as realistic as possible in order to represent actual patients with additional context-specific details. Thus, the response to the case integrates automatic type 1 processes to capture both the effect of intensive training (which relies on the use of heuristics) and affect (regret) to possible acts of omission or commission associated with potentially wrong treatment. The latter was measured using DVAS for assessment of regret in holistic fashion [25] (See also Additional file 1). That is, the regret-related consequences had encompassed all possible harms and benefits envisioned by the respondents. Therefore, we label actually elicited benefits and harms as B_1_ and H_1_.

To activate type 2 deliberations and analytic processes, we provided additional objective data on the management of PE and AML based on the best available evidence in the literature. This was given both in terms of general narrative description of treatment for PE and AML and specific prescriptive statements that “treatment is justified when probability of disease (PE or AML) is sufficiently high for given benefits and harms”. We label the objective benefits and harms as B_2_ and H_2_, respectively.

To keep the scenarios as realistic as possible, benefit and harms parameters were tailored to the case descriptions (PE, AML). Benefits and harms were given for each case (6 vignettes in total). Three vignettes included description of PE and three described AML cases. The three vignettes represented the base-case (intermediate benefits/harms ratio), high-risk (with low benefit/harms ratio resulting in higher threshold in comparison with the base-case), and low-risk (high benefit/harms ratio resulting in lower threshold in comparison with the base-case). In the vignettes, we also provided data on probability of disease (PE or AML relapse, respectively). In addition, when asked “would you give treatment to this patient” in the instruction prior to presenting the first (base-case) vignette, we included a normative statement that “treatment should be given if probability of disease exceeds probability X” where X was derived using B_2_/H_2_ data and referred to the probability of PE and AML, respectively. In PE vignettes, in addition to providing assessment of probability of disease in a base-case vignette, we also included data on the probability of PE in high- and low-risk vignettes (we kept probability of PE in all scenarios at 50%). The intent was to enable type 2 functioning to the maximum possible extent, and to ensure that the observed results are not ascribed to simple error in calculations but rather reflect activation of systematic cognitive processes (see also below). In case of AML, we provided sufficient details from which a physician familiar with treatment of AML could easily deduce high or low probability of relapse (but without including explicit quantitative statements about probability of AML relapse). The intent here was to simulate actual practice where experts typically talk about “high” or “low” risk for relapse, but rarely quantify it. In both cases, we expected to observe the physicians’ behavior according to a threshold model.

Finally, to control for the order of presentation, we randomly presented PE versus AML vignettes. We further randomized the order of presentation to low versus high “threshold” descriptions, and the DVAS anchor used to elicit regret (i.e. we randomized a default slider position at 0% vs. 100%). Thus, all participants were presented all questions related to all vignettes, but the ordering of questions was randomized within the individual participants.

In summary, the manipulated factors were: response stage (initial/final), scenario familiarity (pulmonary embolism/acute myeloid leukemia), and level of threshold (“risk”) according to EUT (high/low B_2_/H_2_ ratio), all manipulated within participants.Figure 1 shows details of the experimental design.

### Statistical analysis

We planned to recruit 40 participants, which is a customary sample size for cognitive psychology experiments. To test our main hypothesis, we postulated the following: if the threshold concept operates, then fewer physicians will give treatment as the threshold probability increases; this is because the physicians will require higher diagnostic certainty to prescribe treatments when threshold level is high. Conversely, as the threshold drops, lower diagnostic certainty is required, and more physicians will prescribe treatment. To assess whether our predictions will bear out, we compared responses to the base-case vignettes with those in which the threshold was higher (“high-risk”, low B_2_/H_2_) or lower (“low-risk”, high B_2_/H_2_) in relation to the base-case scenario. Thus, the main outcome in our study was comparison of a proportion of the physicians who will or will not prescribe treatment in relation to perceived change in the EUT threshold probability. To assess for the difference in responses between base-case and high-risk (low B_2_/H_2_, high threshold) and base-case and low-risk (high B_2_/H_2_, low threshold) scenarios we employed McNemar’s test because of the paired nature of our data [30].

Our secondary outcomes consisted of deriving three thresholds, one for each model (i.e., T_EUT_, T_RG_ and T_DP_) with respect to the given probability of diagnosis of PE and AML relapse, respectively. We postulated that the actual threshold would be lower than the estimated probability of disease for physicians who decided to treat. On the other hand, for physicians who decided not to treat, the threshold will be higher than the estimated probability of disease. We computed the threshold for each participant and assessed whether their decisions to treat or not were in agreement with the particular threshold model. To explain which threshold model can best explain our main results, we assessed the difference in agreement between all three threshold models. Agreement was established if the probability of PE or AML was greater than or equal to threshold and the participant decided to treat or if the probability of PE or AML was less than threshold and the participant decided not to treat. A two-level logit mixed-model was applied which allowed us to account for the correlated multiple responses within each participant for each of the six vignettes. The model was fit using the command meqrlogit in STATA [31].

## Results

A total of 41 consecutively enrolled physicians participated in the web-based survey. Two out of 41 participants were not practicing physicians (1 was a public health professional, and 1 was preparing for residency in internal medicine). Data from these two participants were included in the report as there were no significant differences in the findings when they were removed from the analysis. To ensure that we enrolled a sufficient number of physicians with experience in treating AML, an invitation to participate was first sent to hematology and oncology fellows and the faculty at the USF. After receiving 10 responses, we sent invitations for the survey to all other types of specialties. Details on the demographics of participants and other characteristics are summarized in Table 1. Thirty-eight of the 41 participants (93%) had experience treating PE, while 16 (39%) of physicians had experience with treatment of patients with AML. Both PE and AML vignettes were judged by majority of physicians (79% and 88%, respectively) as realistic examples of real-life clinical situations. Twenty-nine (71%) participants stated that they are familiar with the formal principles of decision analysis (which is based on EUT).

**Table 1:** Participant demographics and experience

**Variable**	**Number of participants (%)**
Overall	41 (100)
Gender	
Male	28 (68)
Female	13 (32)
Age	
Median (Range)	41 (26 to 66)
Area of specialization	
Anesthesiology	2 (5)
Dermatology	1 (2)
Emergency Medicine	1 (2)
Family Medicine	10 (24)
Hematology and Oncology	14 (34)
Internal Medicine	5 (12)
Obstetrics and Gynecology	2 (5)
Otolaryngology	1 (2)
Pediatrics	1 (2)
Urology	2 (5)
Other*	2 (5)
Level of experience	
Resident	10 (24)
Fellow	8 (20)
Attending	23 (56)
Experience treating patients for PE (N = 41)	
None	3 (7)
Fewer than 5 patients	11 (27)
Between 5 and 10 patients	4 (10)
Between 11 and 20 patients	7 (17)
More than 20 patients	16 (39)
PE vignettes similar to experience (N = 38)	
Yes	30 (79)
No	8 (21)
Experience treating patients for AML (N = 41)	
None	25 (61)
Fewer than 5 patients	4 (10)
Between 5 and 10 patients	1 (2)
Between 11 and 20 patients	4 (10)
More than 20 patients	7 (17)
AML vignettes similar to experience (N = 16)	
Yes	14 (88)
No	2 (12)
Understand formal principles of decision analysis (N = 41)	
Yes	29 (71)
No	12 (29)

*One public health and one preparing for residency in internal medicine.

Table 2 shows the results of main analysis. The results are consistent with our main hypothesis: fewer physicians treat as the benefit/harms ratio decreased (i.e. threshold increased) whereas more physicians administered treatment as the benefit/harms ratio went up (and the threshold decreased). A significantly lower proportion of physicians favored treatment in the “high threshold” (high-risk) case compared to the base-case both for PE and AML case vignettes (p < 0.0001). Similarly, a significantly higher proportion of physicians favored treatment in the “low threshold” (low-risk) case compared to the base-case (p < 0.0001) in the AML vignette. However, there were no statistically significant differences in responses between the base-case and “low threshold” case for PE. The reason for this is that, surprisingly, we detected ceiling effects in the PE case: all physicians stated that they would treat the patient in the vignette with high benefit/harm ratio (“low-risk”, “low threshold” vignette) while only one physician would not treat the patient in the base-case vignette. Nevertheless, qualitatively the results went in the same direction providing overall support for our hypotheses. In addition, the results were robust to the sensitivity analyses according to the years of experience, areas of expertise, familiarities with the clinical situation, experience with decision analysis, or order of randomization (see sensitivity analysis in Table two in Additional file 1). Thus, the findings indicate that, relative to base rates, the probability of treatment decreased in the “high threshold” (“high-risk”, low benefit/harm ratio) vignettes, and increased in the “low threshold” (“low-risk”, high benefit/harm ratio) vignettes (except for PE where treatment probability was at ceiling in the base-case and could not increase any further).

**Table 2:** Decision to administer treatment (N = 41)

	**Pulmonary Embolism**					**Acute Myeloid Leukemia**				
**Case**	**Treat (%)**		**No treat (%)**		**p-value**	**Treat (%)**		**No treat (%)**		**p-value**
Base case	40	(98)	1	(2)		27	(66)	14	(34)	
High threshold (“risk”) case	16	(39)	25	(61)	<0.0001	8	(20)	33	(80)	<0.0001
Low (“risk”) threshold case	41	(100)	0	(0)	1	36	(88)	5	(12)	0.012

**Figure 2:**
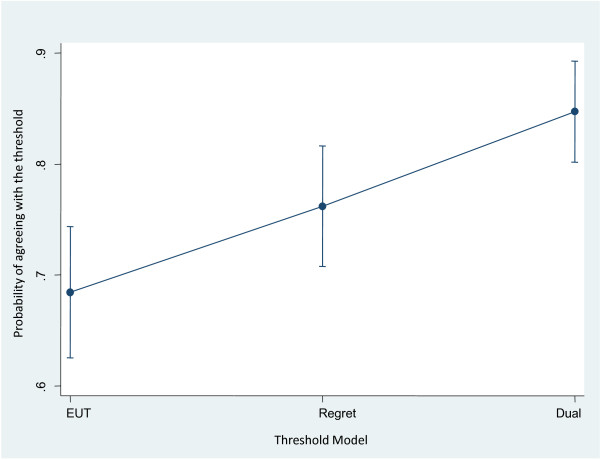
**The predicted probability of the agreeing with threshold for each model**. Dual processing model seems to fit the data best.

The results show that the threshold concept is likely to be operating in clinical practice but does not clarify which threshold model is valid (Table 2). Table 3 shows the threshold value results according to all three threshold models tested (Additional file 2). When compared to the actual treatment recommendations in a pooled mixed model analysis, we found that the regret model was marginally statistically superior to the EUT model [Odds ratio (OR) = 1.49; 95% confidence interval (CI) 1.00 to 2.23; p = 0.06]. The dual-processing model was statistically significantly superior to both the EUT model [OR = 1.75, 95% CI 1.67 to 4.08; p < 0.001] and regret model [OR = 2.61, 95% CI 1.11 to 2.77; p = 0.018]. Figure 2 shows predicted probability of the agreeing with threshold for each model. Thus, the dual-processing threshold model appears to most consistently agree with the observed data.

**Table 3:** Physicians whose decision to administer treatment was in agreement with specific threshold (N = 41)

	**Pulmonary Embolism**							**Acute Myeloid Leukemia**					
	**Agree**	**(%)**	**Disagree**	**(%)**	**EUT versus regret**	**EUT or regret versus dual**		**Agree**	**(%)**	**Disagree**	**(%)**	**EUT versus regret**	**EUT or regret versus dual**
					**p-value**	**p-value**						**p-value**	**p-value**
**Base case**													
EUT	40	(98)	1	(2)		1		27	(66)	14	(34)		0.096
Regret	38	(93)	3	(7)	0.625	0.625		33	(80)	8	(20)	0.146	0.727
Dual	40	(98)	1	(2)				35	(85)	6	(15)		
**High risk case**													
EUT	16	(39)	25	(61)		0.004		8	(20)	33	(80)		<0.001
Regret	31	(76)	10	(24)	0.003	1		25	(61)	16	(39)	<0.001	<0.001
Dual	30	(73)	11	(27)				40	(98)	1	(2)		
**Low risk case**													
EUT	41	(100)	0	(0)		<0.001		36	(88)	5	(12)		0.453
Regret	37	(90)	4	(10)	0.125	0.118		23	(56)	18	(14)	0.011	0.021
Dual	30	(73)	11	(27)				33	(80)	8	(44)		

Note: Agreement was established if the probability of PE or AML was greater than or equal to threshold and the participant decided to treat or the probability of PE or AML was less than threshold and the participant decided not to treat.

## Discussion

In this paper, we provide empirical evidence that physicians appear to make their decisions according to the threshold model. A few empirical studies evaluated if physicians make decisions according to the threshold model [18,19] but none consider putting their results within a specific theoretical framework such as regret or dual processing theories. In this paper, we evaluated three types of threshold models published in the literature so far: 1) EUT [2], 2) regret [3,4], and 3) dual-processing model [6].

Regardless which threshold model can explain physicians’ treatment decisions best, our finding that the threshold model appears to underpin typical clinical decision-making has practical implications for the practice of medicine and medical education. For example, it is estimated that between 30-50% of health care represents waste, mostly due to over-treatment [32]. Furthermore, approximately 80% of all health care expenditures are attributed to physicians’ decisions [33]. If physicians’ do act according to the threshold model, this would mean that every time they perceive that benefits of a treatment substantially outweigh its harms, we can expect that the treatment threshold will predictably drop. The lower the threshold, the lower is the diagnostic certainty required to justify treatment, thereby leading more physicians to prescribe treatment [5,20,21,34]. While this behavior may be rational, it, in turn, will lead to increase in over-treatment [5]. For example, in the baseline case of PE, almost all physicians (98%) would commit to treatment even though probability of PE was only 50%; that is, almost half of patients without PE would be treated unnecessarily. Conversely, the requirement for higher diagnostic certainty may lead to under-treatment. For example, in the high threshold case, only 39% of physicians would give treatment, even though the probability of PE was 50% (Table 2). Thus, depending on the clinical circumstances, both under- and over-treatment do occur in current medical practice and can be explained by the threshold model [4-6]. In general, however, over-treatment dominates the current medical practice in the US [33,35].

Overall, the EUT model predicted the observations with less accuracy compared to regret and dual-processing based models. Although finding that people violate expected utility theory is not new [8,20,21,36-38] it is, however, most interesting that many physicians did not act according to the EUT despite being given prescriptive advice indicating that it may be the most rational approach and regardless of the fact that the majority of them have been exposed to formal principles of decision analysis. The participants satisfied all the criteria for normative response: they had sufficient cognitive ability, high motivation, and appropriate ‘mindware’ i.e., cognitive tools to apply to the task [11], yet they failed to do so. We are not aware of any literature where this has been documented; in fact one lingering question related to the literature about violation of EUT relates to the issue whether the results can be explained by simple computational processing errors in the way people manipulate data on outcomes and probabilities. Our findings show that it is not simple processing errors that led to rejection of EUT. Rather, the results point to the fundamental findings that physicians, like other people [39], do not appear to follow prescriptive EUT as the optimal decision-making framework for medical decision-making. These observations have implications for practice of medicine as influential organizations charged to make clinical recommendations such as the United States Preventive Services Task Force (USPSTF) have increasingly used modeling based on EUT to issue clinical recommendations [40]. The fact that physicians may fail to follow EUT as a basis for decision-making may explain, for example, the vociferous debate that accompanied publication of the USPSTF guidelines on screening mammography [41].

We expected that much of the physicians’ actions are driven by automatic type 1 processes further modified by the contextual details of a given clinical situation. This is the consequence of the way medical education is structured, as the overlearned processes from thousands of hours of training eventually become one’s second nature that serve as the basis for quick, automatic decisions. We found that regret-based B_1_/H_1_ did differ from B_aut_/H_aut_ ratios across presented scenarios (Table 4). This, as stipulated in the Methods, indicates that the contextual characteristics of the cases presented in the vignettes triggered other cognitive mechanisms both along the type 1 (e.g., regret) and type 2 processes.

**Table 4:** Benefit versus harm ratio based on type 1 response*

**Variable**		**n**	**Mean**	**Min**	**Median**	**Max**
**PE**	**B**_**aut**_**/H**_**aut**_	40	4.33	.6	3.00	25.00
**Base case**	**B**_**1**_**/H**_**1**_	40	6.28	0.75	3.18	49.50
**Low risk**	**B**_**1**_**/H**_**1**_	39	12.46	0.66	5.26	100.00
**High risk**	**B**_**1**_**/H**_**1**_	41	1.76	0.05	0.98	18.80
**AML**	**B**_**aut**_**/H**_**aut**_	41	2.29	0.43	2.00	10.00
**Base case**	**B**_**1**_**/H**_**1**_	41	1.55	0.00	1.00	7.07
**Low risk**	**B**_**1**_**/H**_**1**_	39	4.39	0.00	1.94	22.50
**High risk**	**B**_**1**_**/H**_**1**_	40	0.70	0.00	0.50	3.00

Abbreviations: *B*_*aut*_/*H*_*aut*_ assessment of benefit/harms ratio based on automatic, quick response, *B*_*1*_/*H*_*1*_-type 1 response driven by regret, *PE* pulmonary embolism, *AML* acute myeloid leukemia, *low “risk”* low threshold, *high “risk”* high threshold clinical decisions. [*Note that type 2 responses that relied on single values, fixed B_2_/H_2_ ratios precluding direct statistical comparisons with B_aut_/H_aut_. However, the values of B_2_/H_2_ differed considerably from B_aut_/H_aut_ (from 1 to 10 in PE case, and 2 to 0.33 in AML case) consistent with a notion that the B_aut_/H_aut_ estimates did not solely drive the decision-making (see Discussion)].

Our model has certain limitations. Although our data do suggest physicians’ decision-making is more compatible with dual processing model than with the EUT or a simple regret model (Figure 2), our sample size was not large enough to provide more conclusive support in favor of dual processing model in each specific scenario (Table 3). This was the main limitation of our study. Nevertheless, theoretically, the results fit dual processing theories well, because treatment of PE is familiar to most physicians and AML is not. Novel problems trigger type 2 processing; so, for the relatively unfamiliar AML scenarios, dual processing (which takes both type 1 and type 2 processes into account) has predictive advantage. We should, of course, note that our results do not exclude the possibility that some people do act according to either EUT or regret model (Figure 2). In addition, as noted earlier, there are many dual-processing theories [38] and we evaluated a specific dual-processing model that is applicable to single-point clinical decisions such as those described in the vignettes [6] (see Additional file 1). A different model and experimental design would be needed for testing the way physicians make repeated decisions.

Our results also hold promise in medical education. We demonstrated that, at least in some circumstances, physicians do act according to the threshold model. Therefore, all medical curricula should include the teaching the threshold model(s). Although, on average, dual processing model has performed better, we believe that all 3 models should be taught because they collectively take into account the most salient features of human decision-making (assessment of the likelihood of disease and benefit/harms ratio), which are determined by both type 1 (fast, intuitive) and type 2 (slow, deliberative) reasoning processes. In addition, as outlined above, these descriptive models may conceivably be used in prescriptive fashion under some circumstances. For example, in circumstances where our affect plays a key role in the way we feel the consequences of benefits and harms, we may rely on regret approach. Conversely, where empirical evidence on benefits and harms is a driver of decision-making, then application of EUT may still be more suitable. However, we suspect that integration of both approaches, regret- and EUT-based, into dual processing model will be useful to most users. The details of how this integration may work is beyond a scope of this paper, but is sketched in [6].

Certainly, we need confirmatory and larger studies to reproduce (or refute) our results. While we found that the vignettes were judged by the vast majority of physicians as realistic examples of real-life clinical cases, it is still possible that different scenarios and different wording may elicit different responses. Although including realistic and familiar scenarios can be deemed as one of the strengths of our analysis, it has generated some analytical problems, as outlined above. Therefore, the future research should include larger studies with relatively less familiar, but still realistic-case vignettes.

## Conclusions

We find that physicians appear to make treatment decisions according to the threshold model. Furthermore, physicians’ decision-making seems more compatible with the dual processing model than with either EUT or a simple regret model. While larger confirmatory studies are needed to affirm our results, the findings of this study may help improve our understanding of clinical decision making under diagnostic uncertainty and may be helpful in development of medical education curricula and practice guidelines.

## Abbreviations

EUT: Expected utility theory; T_EUT_: Expected utility theory based threshold; T_RG_: Regret-based threshold; T_DP_: Dual-processing theory based threshold; B/H: Benefit to harm ratio; PE: Pulmonary embolism; AML: Acute myeloid leukemia; B_aut_: Automatic benefits assessment; H_aut_: Automatic harms assessment; B_1_: Initial type 1 benefits assessment; H_1_: Initial type 1 harms assessment; DVAS: Dual Visual Analog Scale; B_2_: Objective benefits assessment; H_2_: Objective harms assessment; OR: Odds ratio; CI: Confidence interval.

## Competing interests

None of the authors have any financial competing interests to disclose.

## Authors’ contributions

BD was responsible for concept and design of the study, analysis and interpretation of data, and drafting the manuscript. SE contributed to study design, analysis and interpretation of data, and revision of the manuscript for critically important intellectual content. TR contributed to study design, acquisition of data, analysis and interpretation of data, and revision of the manuscript for critically important intellectual content. IH contributed to analysis and interpretation of data and revision of the manuscript for critically important intellectual content. BM contributed to analysis and interpretation of data and revision of the manuscript for critically important intellectual content. AT contributed to study design, data acquisition, and revision of the manuscript for critically important intellectual content. AK contributed to study design, interpretation of data, and drafting of the manuscript. JB contributed to concept and study design and revision of the manuscript for critically important intellectual content. ST contributed to acquisition of data, and revision of the manuscript for critically important intellectual content. JCB contributed to study design, analysis and interpretation of data, and revision of the manuscript for critically important intellectual content. All authors read and approved the final manuscript.

## Pre-publication history

The pre-publication history for this paper can be accessed here:

http://www.biomedcentral.com/1472-6947/14/47/prepub

## Supplementary Material

Additional file 1 The survey. Click here for file

Additional file 2: Table S1 Sensitivity analysis. Click here for file

## References

[B1] Pauker SG Kassirer J The threshold approach to clinical decision making N Engl J Med 1980 302 1109 1117 10.1056/NEJM198005153022003 7366635

[B2] Pauker SG Kassirer JP Therapeutic decision making: a cost benefit analysis N Engl J Med 1975 293 229 234 10.1056/NEJM197507312930505 1143303

[B3] Djulbegovic B Hozo I Schwartz A McMasters K Acceptable regret in medical decision making Med Hypotheses 1999 53 253 259 10.1054/mehy.1998.0020 10580533

[B4] Hozo I Djulbegovic B When is diagnostic testing inappropriate or irrational? Acceptable regret approach Med Decis Making 2008 28 4 540 553 10.1177/0272989X08315249 18480041

[B5] Hozo I Djulbegovic B Will insistence on practicing medicine according to expected utility theory lead to an increase in diagnostic testing? Med Decis Making 2009 29 320 322 10.1177/0272989X09334370

[B6] Djulbegovic B Hozo I Beckstead J Tsalatsanis A Pauker SG Dual processing model of medical decision-making BMC Med Inform Decis Mak 2012 12 1 94 10.1186/1472-6947-12-94 22943520 PMC3471048

[B7] Kahneman D Maps of bounded rationality: psychology for behavioral economics American Economic Review 2003 93 1449 1475 10.1257/000282803322655392

[B8] Kahnemen D Thinking fast and slow 2011 New York: Farrar, Straus and Giroux

[B9] Evans JSTBT Hypothethical thinking. Dual processes in reasoning and judgement 2007 New York: Psychology Press: Taylor and Francis Group

[B10] Stanovich KE West RF Individual differences in reasoning: implications for the rationality debate? Behav Brain Sci 2000 23 645 726 10.1017/S0140525X00003435 11301544

[B11] Stanovich KE Rationality and the Reflective Mind 2011 Oxford: Oxford University Press

[B12] Croskerry P Clinical cognition and diagnostic error: applications of a dual process model of reasoning Adv Health Sci Educ Theory Pract 2009 14 Suppl 1 27 35 19669918 10.1007/s10459-009-9182-2

[B13] Croskerry P A universal model of diagnostic reasoning Acad Med 2009 84 8 1022 1028 10.1097/ACM.0b013e3181ace703 19638766

[B14] Croskerry P Abbass A Wu AW Emotional influences in patient safety J Patient Saf 2010 6 4 199 205 10.1097/PTS.0b013e3181f6c01a 21500605

[B15] Croskerry P Nimmo GR Better clinical decision making and reducing diagnostic error J R Coll Physicians Edinb 2011 41 2 155 162 10.4997/JRCPE.2011.208 21677922

[B16] Slovic P Finucane ML Peters E MacGregor DG Risk as analysis and risk as feelings: some thoughts about affect, reason, risk, and rationality Risk Anal 2004 24 2 311 322 10.1111/j.0272-4332.2004.00433.x 15078302

[B17] Tversky A Kahneman D The framing of decisions and the psychology of choice Science 1981 211 4481 453 458 10.1126/science.7455683 7455683

[B18] Basinga P Moreira J Bisoffi Z Bisig B Van den Ende J Why are clinicians reluctant to treat smear-negative tuberculosis? An inquiry about treatment thresholds in Rwanda Med Decis Making 2007 27 1 53 60 10.1177/0272989X06297104 17237453

[B19] Eisenberg JM Hershey JC Derived thresholds: determining the diagnostic probabilities at which clinicians initiate testing and treatment Med Decis Making 1983 3 155 168 10.1177/0272989X8300300203 6415358

[B20] Moreira J Alarcon F Bisoffi Z Rivera J Salinas R Menten J Duenas G Van den Ende J Tuberculous meningitis: does lowering the treatment threshold result in many more treated patients? Trop Med Int Health 2008 13 1 68 75 10.1111/j.1365-3156.2007.01975.x 18291004

[B21] Tuyisenge L Ndimubanzi CP Ndayisaba G Muganga N Menten J Boelaert M Van den Ende J Evaluation of latent class analysis and decision thresholds to guide the diagnosis of pediatric tuberculosis in a Rwandan reference hospital Pediatr Infect Dis J 2010 29 e11 e18 10.1097/INF.0b013e3181c61ddb 19935116

[B22] Zeelenberg M Pieters R A theory of regret regulation 1.1 J Consumer Psychol 2007 17 29 35 10.1207/s15327663jcp1701_6

[B23] Zeelenberg M Pieters R A Theory of Regret Regulation 1.0 J Consumer Psychol 2007 17 1 3 18 10.1207/s15327663jcp1701_3

[B24] Jannis IL Mann L Decision Making. A psychological Analysis of Conflict, Choice, and Committment 1977 London: The Free Press

[B25] Tsalatsanis A Hozo I Vickers A Djulbegovic B A regret theory approach to decision curve analysis: A novel method for eliciting decision makers’ preferences and decision-making BMC Med Inform Decis Mak 2010 10 1 51 10.1186/1472-6947-10-51 20846413 PMC2954854

[B26] Evans JSTBT The heuristic-analytic theory of reasoning: extension and evaluation Psychon Bull Rev 2006 13 378 395 10.3758/BF03193858 17048720

[B27] Evans JSTBT Thinking Twice. Two Minds in One Brain 2010 Oxford: Oxford University Press

[B28] Mukherjee K A dual system model of preferences under risk Psychol Rev 2010 177 1 243 255 20063971 10.1037/a0017884

[B29] Evans JSTBT Dual-process theories of reasoning: Contemporary issues and developmental applications Developmental Review 2011 31 86 102 10.1016/j.dr.2011.07.007

[B30] McNemar Q Note on the sampling error of the difference between correlated proportions or percentages Psychometrika 1947 12 2 153 157 10.1007/BF02295996 20254758

[B31] STATA Corporation STATA, ver. 12 2010 College Station, TX

[B32] Berwick DM Hackbarth AD Eliminating Waste in US Health Care JAMA 2012 307 14 1513 1516 10.1001/jama.2012.362 22419800

[B33] Cassel CK Guest JA Choosing Wisely JAMA 2012 307 17 1801 1802 10.1001/jama.2012.476 22492759

[B34] Van den Ende J Moreira J Tuyisenge L Bisoffi Z An Inquiry About Clinicians’ View of the Distribution of Posttest Probabilities: Possible Consequences for Applying the Threshold Concept Med Decis Making 2013 33 2 136 8 10.1177/0272989X12448681 22647831

[B35] Djulbegovic B Paul A From efficacy to effectiveness in the face of uncertainty: indication creep and prevention creep JAMA 2011 305 19 2005 2006 21586716 10.1001/jama.2011.650

[B36] Kahneman D Tversky A “Prospect theory”: an analysis of decion under risk Econometrica 1979 47 263 291 10.2307/1914185

[B37] Kahneman D Wakker PP Sarin RK Back to Bentham? Explorations of Experienced Utility Quarterly Journal of Economics 1997 112 375 405 10.1162/003355397555235

[B38] Reyna VF A new intuitionism: Meaning, memory, and development in Fuzzy-Trace Theory Judgment and Decision Making 2012 7 3 332 359 PMC4268540 25530822

[B39] Elqayam S Grounded rationality: descriptivism in epistemic context Synthese 2012 189 39 49 10.1007/s11229-012-0153-4

[B40] US Preventive Service Task Force Screening for Breast Cancer: U.S. Preventive Services Task Force Recommendation Statement Ann Intern Med 2009 151 716 726 19920272 10.7326/0003-4819-151-10-200911170-00008

[B41] Editors When Evidence Collides With Anecdote, Politics, and Emotion: Breast Cancer Screening Ann Intern Med 2010 152 8 531 532 20157099 10.7326/0003-4819-152-8-201004200-00210

