# Supplementary material for: How do physicians decide to treat: an empirical evaluation of the threshold model
Source: BMC Med Inform Decis Mak. 2014 Jun 5;14:47. doi: 10.1186/1472-6947-14-47 (PMC4055375; doi:10.1186/1472-6947-14-47)
Supplement: Additional file 1 — The survey. [file 1472-6947-14-47-S1.docx]

**Appendix 1 (Survey)**

(edited to facilitate better understanding; the actual vignettes were administered randomly. The survey also first began by collecting data on demographics- see Table 1)

**Case #1: Pulmonary Embolism**

Pulmonary embolism (PE) is a serious condition which may result in death. Anticoagulants are used to reduce the risk of blood clot expansion; however, they are associated with an increased chance of serious bleeding.

**Please estimate the benefit to harm ratio related to administering an anticoagulant (heparin)?**

*(Note: a ratio of 1:1 indicates that a patient is equally likely to benefit as to be harmed by the treatment, a ratio of 2:1 indicates that a patient is twice as likely to benefit from the treatment as be harmed by it, and a ratio of 1:2 indicates that the patient is twice as likely to be harmed by the treatment than to benefit from it)*

_________:_________

Heparin is a highly effective treatment for pulmonary embolism (PE) associated with a relative risk reduction of death from PE by about 80% in comparison with no treatment. This converts into an absolute reduction in mortality (net benefits) of 20%. However, anticoagulants are also associated with a significant risk for life-threatening bleeding with the net harms ranging from a low of 1% to a high of 10%.

The net benefit to harm ratio associated with the use of anticoagulants in patients with PE ranges from (20:1) in the best case clinical scenario to (2:1) in the worst case clinical scenario. Treatment with anticoagulants is justified when probability of PE is sufficiently high for a given benefits and harms. This means that anticoagulants (heparin) should be recommended to any patient with the probability of PE greater than 5% in the best case scenario and 33% in the worst case scenario.

**PE– base case**

A 78 year-old male underwent hip replacement surgery 2 weeks ago and complains of pain in the left lower extremity. His heart rate is 100 beats/min. His hemoglobin level is 14 g/dL and his creatinine level is 1.4 mg/dL. Given these indicators, the probability that the patient has pulmonary embolism (PE) is 50%. While you would like to initiate treatment, you also estimated that the patient has around 3% risk of bleeding. No other diagnostic tests are available to you.

The benefit to harm ratio for this patient is 3:1.

As no decision can be absolutely accurate, it is always possible that you recommended anticoagulant therapy to a patient who shouldn't have received treatment or that you failed to recommend anticoagulant therapy to a patient who should have been treated. As a result you may feel regret.

On a scale of 0 to 100, where 0 indicates no regret and 100 indicates the maximum regret, how would you rate the level of your regret if you did not administer the anticoagulant therapy to this patient, which resulted in pulmonary embolism and death?


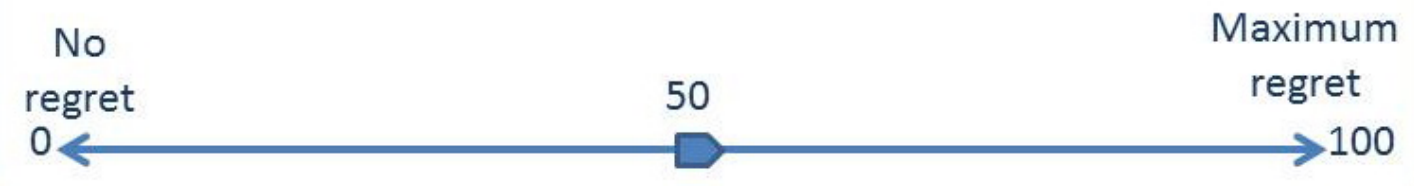


On a scale of 0 to 100, where 0 indicates no regret and 100 indicates the maximum regret, how would you rate the level of your regret if you unnecessarily administered the anticoagulant therapy to this patient, which resulted in massive bleeding and death?


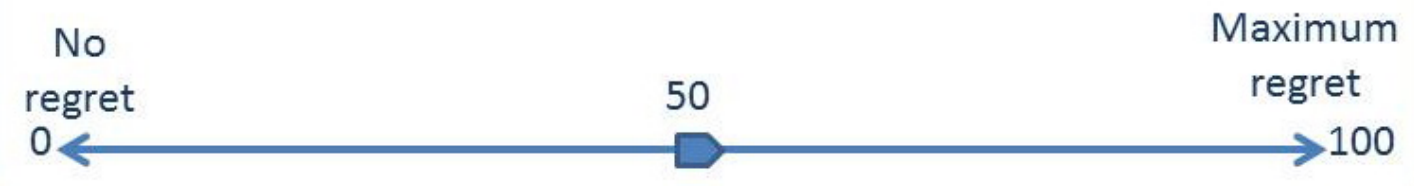


Theoretically, benefits of treatment outweigh its harms for this patient. That is, if the probability that the patient has PE is greater than 25%, the patient should be given anticoagulants.

**Would you recommend anticoagulant therapy to this patient?**

**YES NO**

You selected 'NO' to recommending anticoagulant therapy in this patient.

How certain do you need to be in the diagnosis of pulmonary embolism (PE) before administering anticoagulants? That is, what is probability of PE at or above which you would give anticoagulants (heparin) (0 to 100%) to this patient?


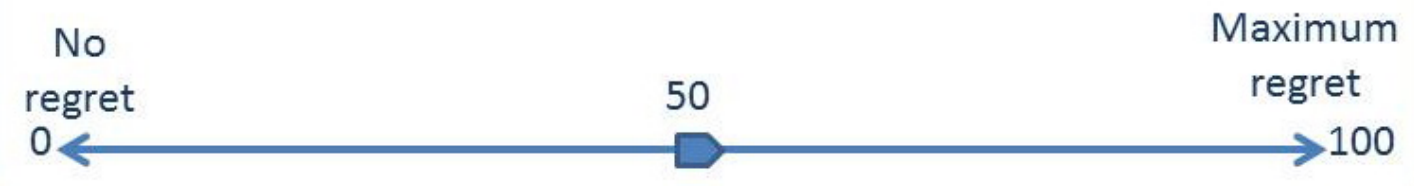

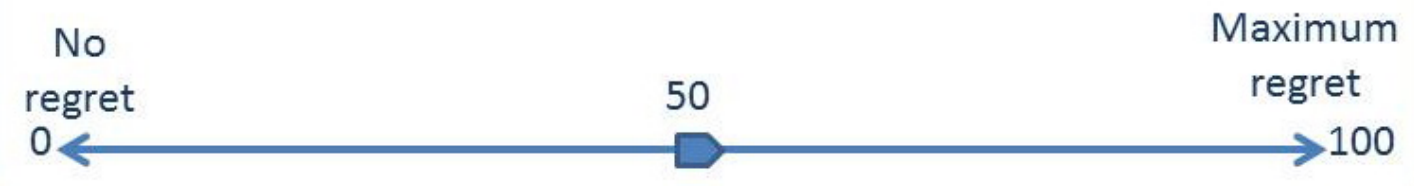


**PE – High risk case (Case A)**

A 82 year-old female was diagnosed with cancer 3 months ago. She is currently in the hospital following a recent major bleeding event. Her hemoglobin level is 9 g/dL and her creatinine level is 1.7 mg/dL. Her heart rate is 80 beats/min. The patient has a history of deep venous thrombosis and presents with sudden shortness of breath. You suspect the patient has a 50% probability of pulmonary embolism (PE). Prior to recommending anticoagulant therapy with heparin, you assess the patient’s risk of bleeding. Given the indicators, you suspect the patient’s probability of bleeding is 10%. No other diagnostic tests are available to you.

The benefit to harm ratio of administering anticoagulants (heparin) to this patient is 1:1.

As no decision can be absolutely accurate, it is always possible that you recommended anticoagulant therapy to a patient who shouldn't have received treatment or that you failed to recommend anticoagulant therapy to a patient who should have been treated. As a result you may feel regret.

On a scale of 0 to 100, where 0 indicates no regret and 100 indicates the maximum regret, how would you rate the level of your regret if you did not administer the anticoagulant therapy to this patient, which resulted in pulmonary embolism and death?


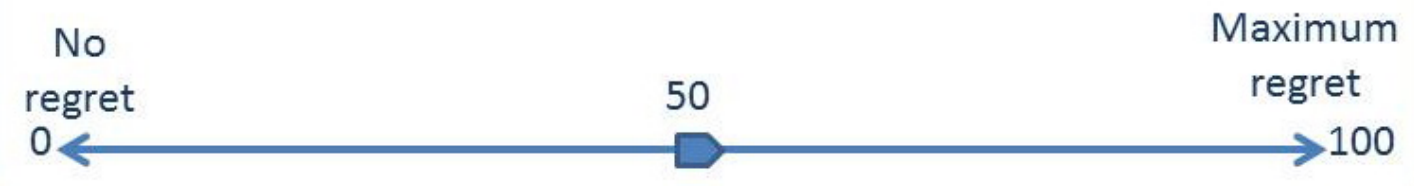


On a scale of 0 to 100, where 0 indicates no regret and 100 indicates the maximum regret, how would you rate the level of your regret if you unnecessarily administered the anticoagulant therapy to this patient, which resulted in massive bleeding and death?


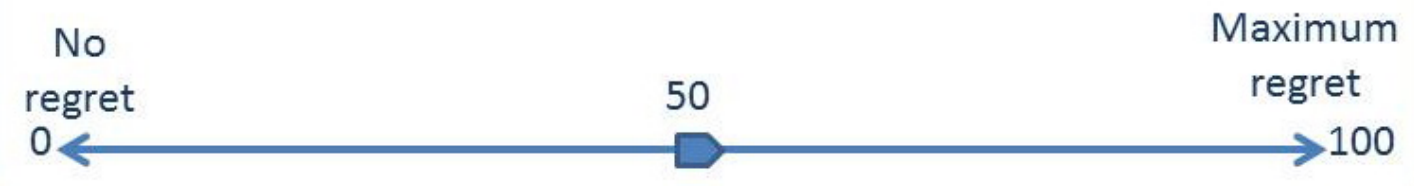


**Would you recommend anticoagulant therapy to this patient?**

**YES NO**

You selected 'NO' to recommending anticoagulant therapy in this patient.

How certain do you need to be in the diagnosis of pulmonary embolism (PE) before administering anticoagulants? That is, what is probability of PE at or above which you would give anticoagulants (heparin) (0 to 100%) to this patient?


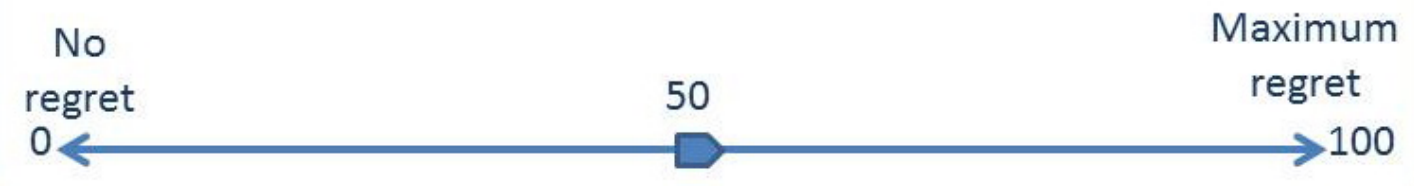

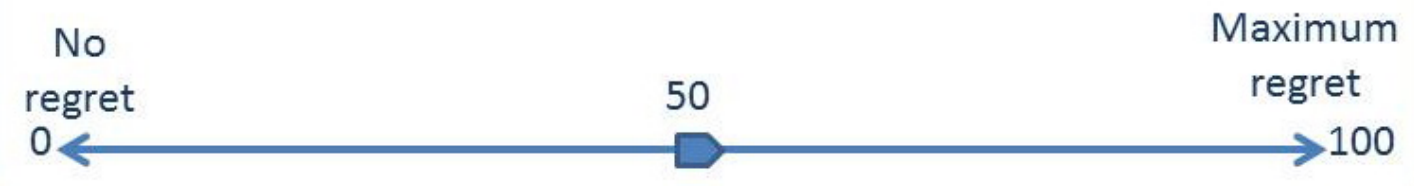


**PE – Low risk case (Case B)**

A 25 y/o male fractured his left leg 1 week ago complains of a new pain in his left leg and shortness of breath. His hemoglobin level is 16 g/dL. His creatinine level is 1.0 mg/dL. Heart rate is 88 beats per min. Given this information, you conclude his probability of having pulmonary embolism is 50%. Prior to recommending anticoagulant therapy, you evaluate the patient for risk of bleeding. The patient’s risk of bleeding is around 1% or less. No other diagnostic tests are available to you.

The benefit to harm ratio for this patient is 10:1.

As no decision can be absolutely accurate, it is always possible that you recommended anticoagulant therapy to a patient who shouldn't have received treatment or that you failed to recommend anticoagulant therapy to a patient who should have been treated. As a result you may feel regret.

On a scale of 0 to 100, where 0 indicates no regret and 100 indicates the maximum regret, how would you rate the level of your regret if you did not administer the anticoagulant therapy to this patient, which resulted in pulmonary embolism and death?


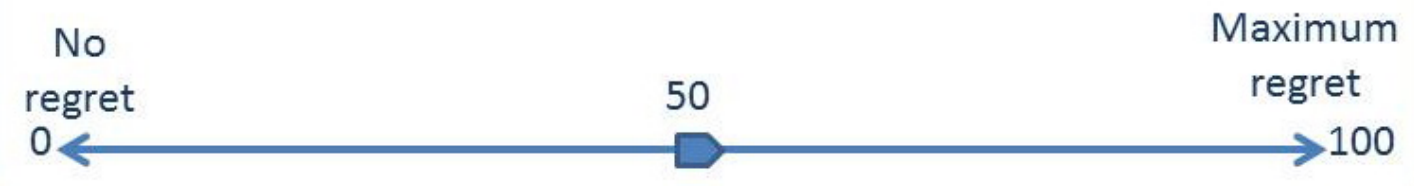


On a scale of 0 to 100, where 0 indicates no regret and 100 indicates the maximum regret, how would you rate the level of your regret if you unnecessarily administered the anticoagulant therapy to this patient, which resulted in massive bleeding and death?


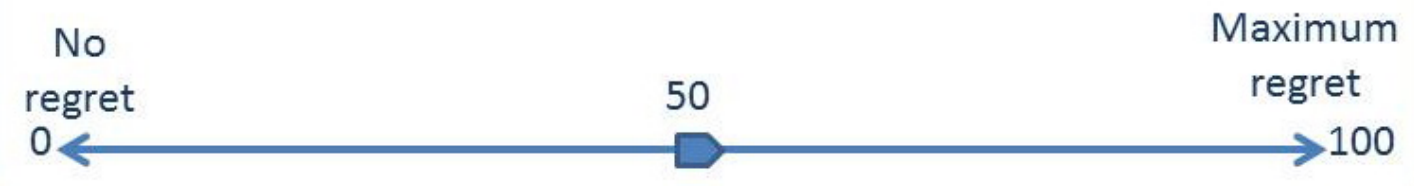


**Would you recommend anticoagulant therapy to this patient?**

**YES NO**

You selected 'NO' to recommending anticoagulant therapy in this patient.

How certain do you need to be in the diagnosis of pulmonary embolism (PE) before administering anticoagulants? That is, what is probability of PE at or above which you would give anticoagulants (heparin) (0 to 100%) to this patient?


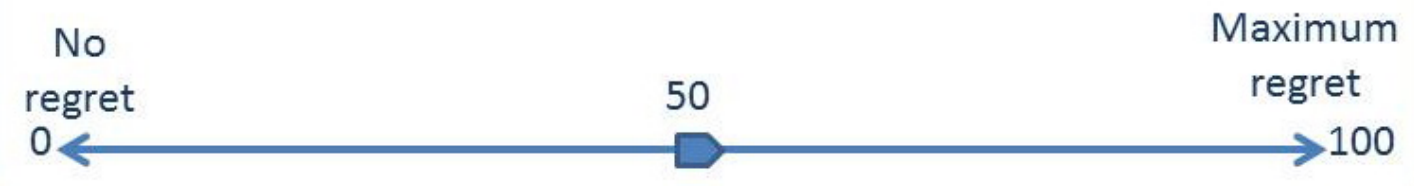

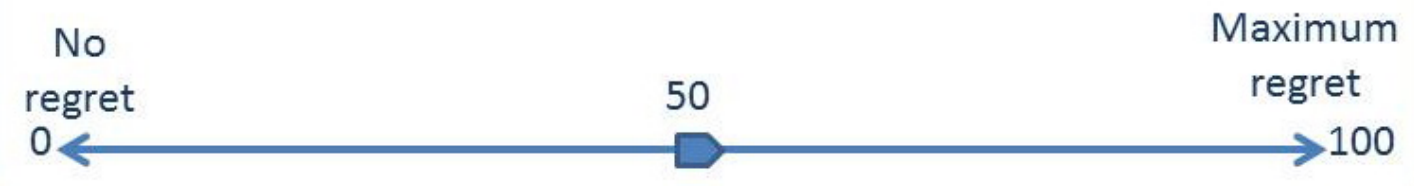


**Case #2: Acute Myeloid Leukemia**

Acute myeloid leukemia (AML) is a life-threatening disease which can be treated by either allogeneic stem cell transplantation (allo-SCT) or chemotherapy. Allo-SCT can cure more patients than chemotherapy; however, allo-SCT is associated with higher treatment related mortality than chemotherapy.

**Please estimate the benefit to harm ratio related to recommending allo-SCT in comparison to chemotherapy?**

*(Note: a ratio of 1:1 indicates that a patient is equally likely to benefit as to be harmed by the treatment, a ratio of 2:1 indicates that a patient is twice as likely to benefit from the treatment as be harmed by it, and a ratio of 1:2 indicates that the patient is twice as likely to be harmed by the treatment than to benefit from it)*

_________:_________

Allogeneic stem cell transplantation (allo-SCT) is the recommended treatment for acute myeloid leukemia (AML) patients with intermediate or poor-risk disease. Allo-SCT is associated with a 12% better leukemia-free survival when compared to chemotherapy (53% with allo-SCT versus 41% with chemotherapy). However, allo-SCT is also associated with higher treatment related mortality that can range from as low as 6% to as high as 36%. The net benefit to harm ratio associated with the use of allo-SCT in patients with AML can range from 2:1 (best case scenario) to 1:3 (worst case scenario).

Treatment is justified when probability of AML is sufficiently high for a given benefits and harms. This means that allo-SCT should be recommended to any patient with a risk of AML relapse greater than 33% (best case) and 75% (worst case scenario).

**AML – base case**

A 45 year-old man was diagnosed with acute myeloid leukemia. His cytogenetics indicate translocation t(9;11) consistent with intermediate-risk disease. After induction chemotherapy his disease has achieved complete response. The patient has no other co-morbidities. You are considering recommending allogeneic stem cell transplantation (allo-SCT) (versus chemotherapy) to cure his disease. The patient has a perfect HLA match from his brother (10 of 10). Allo-SCT will lead to cure in 12% of cases like this patient; cure is not possible with chemotherapy. However, allo-SCT is associated with 24% of treatment-related mortality in patients with these clinical characteristics. Therefore, benefit to harms ratio of allo-SCT versus chemotherapy is 1:2.

As no decision can be absolutely accurate, it is always possible that you recommended allo-SCT to a patient who shouldn't have received allo-SCT or that you failed to recommend allo-SCT to a patient who should have received it. As a result you may feel regret.

On a scale of 0 to 100, where 0 indicates no regret and 100 indicates the maximum regret, how would you rate the level of your regret if you failed to recommend allo-SCT to this patient and this resulted in acute myeloid leukemia relapse and death?


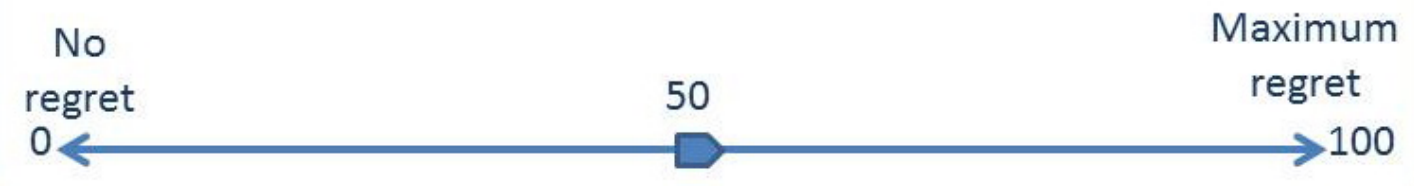


On a scale of 0 to 100, where 0 indicates no regret and 100 indicates the maximum regret, how would you rate the level of your regret if you recommended allo-SCT to this patient, who died as a result of treatment?


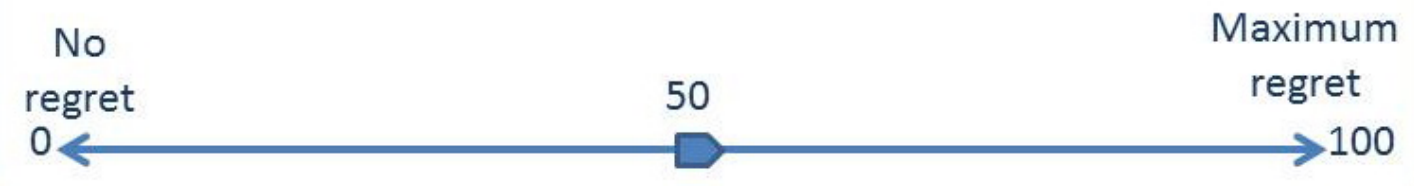


Theoretically, benefits of treatment in a relation to its harms for this patient are such that allo-SCT should be given if the probability that the patient will have acute myeloid leukemia relapse is higher than 66%.

**Would you recommend an allogeneic stem cell transplantation for this patient?**

**YES NO**

You selected 'NO' to recommending allo-SCT to this patient.

How likely does acute myeloid leukemia (AML) relapse need to be for you to recommend allogeneic stem cell transplantation (allo-SCT) to this patient? That is, at or above which probability of relapse (0 to 100%) would you recommend that this patient undergo allo-SCT?


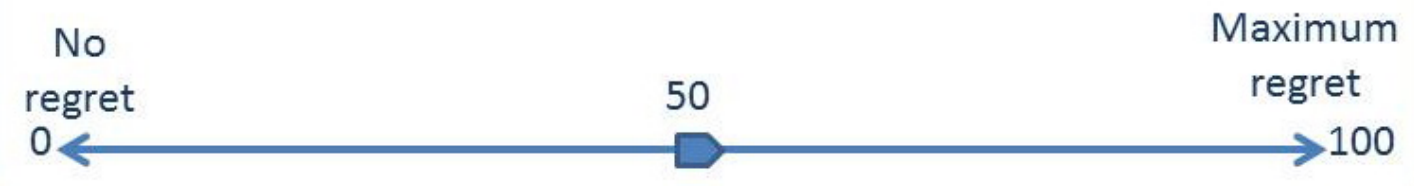

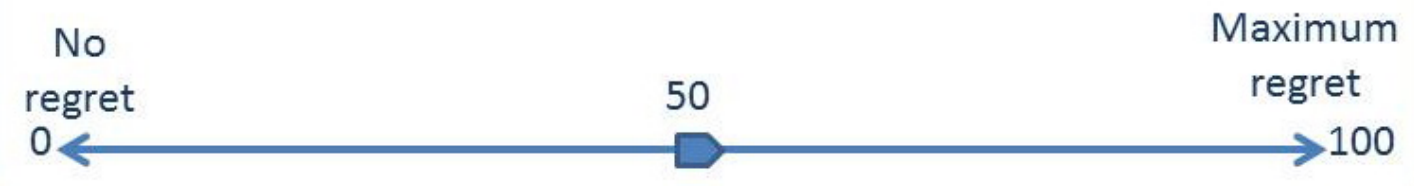


**AML – High risk case (Case A)**

A 72 year-old woman was diagnosed with acute myeloid leukemia. Her cytogenetics indicate -7 deletion consistent with a poor-risk disease. After induction chemotherapy her disease has achieved complete response. The patient has well controlled hypertension and type 2 diabetes, but no other comorbidities. You are considering recommending allogeneic stem cell transplantation (allo-SCT) (versus chemotherapy) to cure her disease. The patient has a perfect HLA match from her sister (10 of 10). Allo-SCT will lead to cure in 12% patients like this; cure is not possible with chemotherapy. However, allo-SCT is associated with 36% treatment-related mortality in patients with these clinical characteristics. Therefore, benefit to harms ratio of allo-SCT versus chemotherapy is 1:3.

As no decision can be absolutely accurate, it is always possible that you recommended allo-SCT to a patient who shouldn't have received allo-SCT or that you failed to recommend allo-SCT to a patient who should have received it. As a result you may feel regret.

On a scale of 0 to 100, where 0 indicates no regret and 100 indicates the maximum regret, how would you rate the level of your regret if you failed to recommend allo-SCT to this patient and this resulted in acute myeloid leukemia relapse and death?


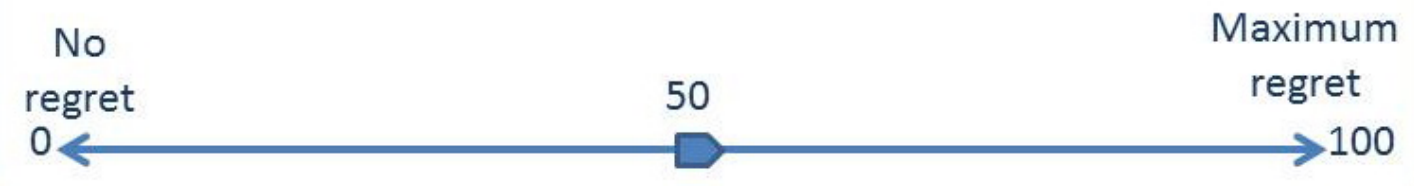


On a scale of 0 to 100, where 0 indicates no regret and 100 indicates the maximum regret, how would you rate the level of your regret if you recommended allo-SCT to this patient, who died as a result of treatment?


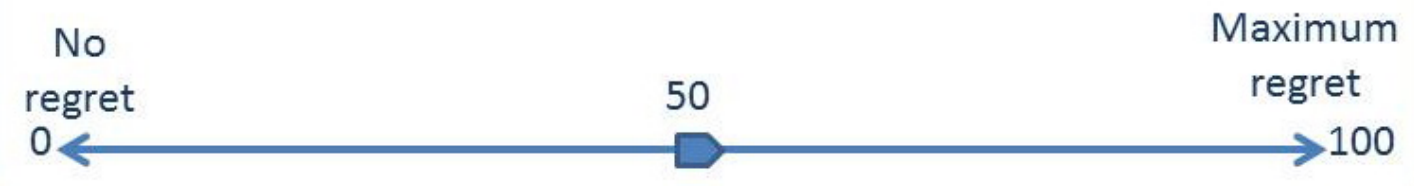


**Would you recommend an allogeneic stem cell transplantation for this patient?**

**YES NO**

You selected 'NO' to recommending allo-SCT to this patient.

How likely does acute myeloid leukemia (AML) relapse need to be for you to recommend allogeneic stem cell transplantation (allo-SCT) to this patient? That is, at or above which probability of relapse (0 to 100%) would you recommend that this patient undergo allo-SCT?


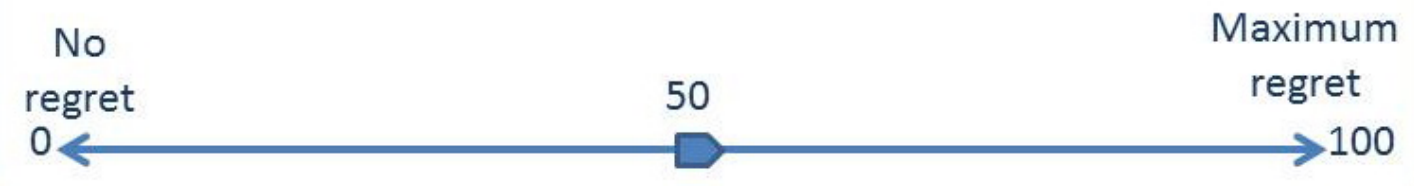

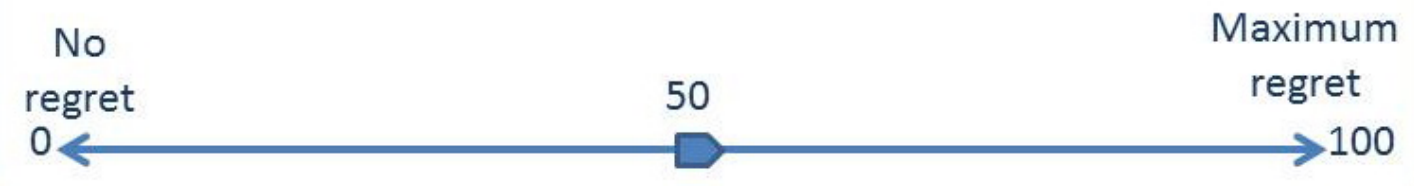


**AML – Low risk case (Case B)**

A 25 year-old man was diagnosed with acute myeloid leukemia. His cytogenetics are normal. The patient is otherwise in excellent condition and has tolerated induction treatment with no major complications. After induction chemotherapy his disease has achieved complete response. You are considering recommending allogeneic stem cell transplantation (allo-SCT) (versus chemotherapy) to cure his disease. The patient has a perfect HLA match from his brother (10 of 10). Allo-SCT will lead to cure in 12% of cases like this patient; cure is not possible with chemotherapy. However, allo-SCT is associated with 6% treatment-related mortality in patients with these clinical characteristics. Therefore, benefit to harms ratio of allo-SCT versus chemotherapy is 2:1.

As no decision can be absolutely accurate, it is always possible that you recommended allo-SCT to a patient who shouldn't have received allo-SCT or that you failed to recommend allo-SCT to a patient who should have received it. As a result you may feel regret.

On a scale of 0 to 100, where 0 indicates no regret and 100 indicates the maximum regret, how would you rate the level of your regret if you failed to recommend allo-SCT to this patient and this resulted in acute myeloid leukemia relapse and death?


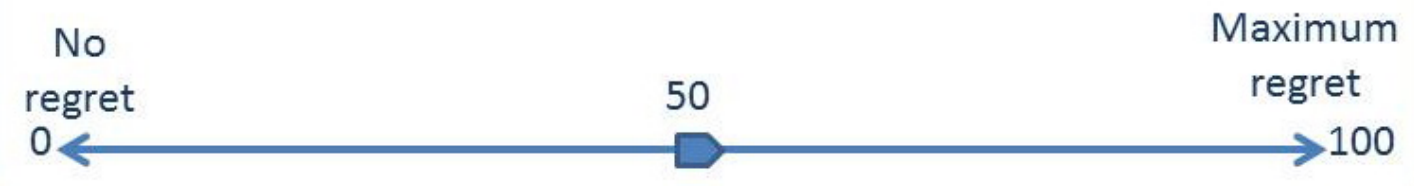


On a scale of 0 to 100, where 0 indicates no regret and 100 indicates the maximum regret, how would you rate the level of your regret if you recommended allo-SCT to this patient, who died as a result of treatment?


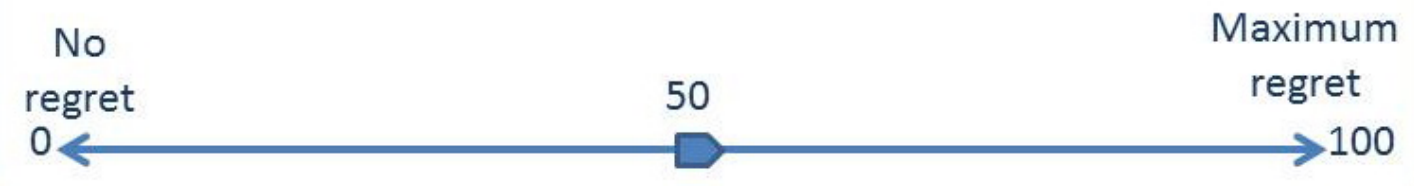


**Would you recommend an allogeneic stem cell transplantation for this patient?**

**YES NO**

You selected 'NO' to recommending allo-SCT to this patient.

How likely does acute myeloid leukemia (AML) relapse need to be for you to recommend allogeneic stem cell transplantation (allo-SCT) to this patient? That is, at or above which probability of relapse (0 to 100%) would you recommend that this patient undergo allo-SCT?


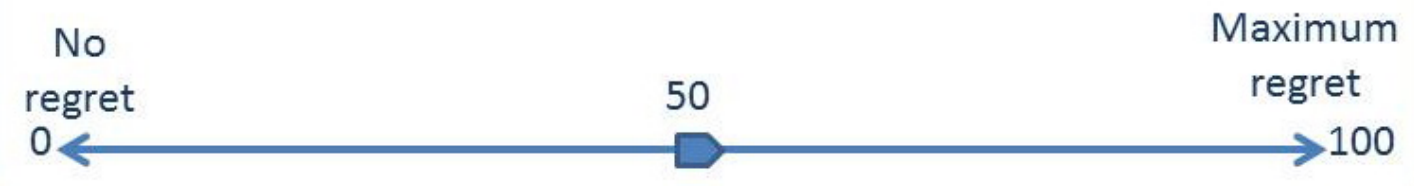

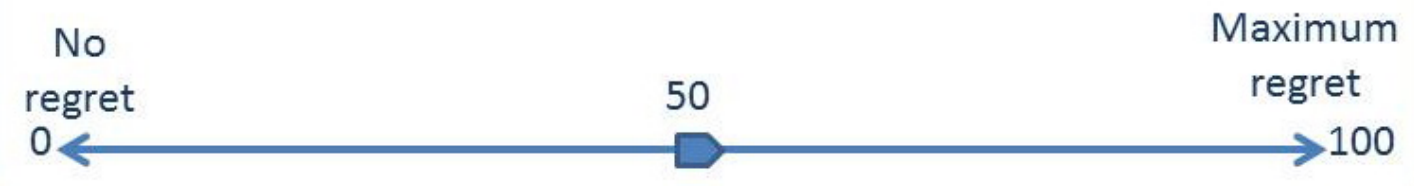


Please feel free to provide us with any comments regarding this survey.

Have you ever treated a patient with pulmonary embolism with anticoagulants (heparin)?

**YES NO**

If yes, approximately how many patients with pulmonary embolism did you treat with anticoagulants?

Less than 5

5 to 10

11 to 20

More than 20

Did any of the pulmonary embolism cases in the vignettes resemble your actual case?

**YES NO**

Have you ever been involved in making recommendations related to administration of allogeneic stem cell transplant to a patient with acute myeloid leukemia?

**YES NO**

If yes, approximately how many patients have you been involved in making recommendations related to administration of allogeneic stem cell transplant for acute myeloid leukemia?

Less than 5

5 to 10

11 to 20

More than 20

Did any of the acute myeloid leukemia cases in the vignettes resemble your actual case?

**YES NO**

Do you consider yourself as someone who understands the formal principles of decision analysis?

**YES NO**

Thank you for participating in our study.
