# Supplementary material for: How do physicians decide to treat: an empirical evaluation of the threshold model
Source: BMC Med Inform Decis Mak. 2014 Jun 5;14:47. doi: 10.1186/1472-6947-14-47 (PMC4055375; doi:10.1186/1472-6947-14-47)
Supplement: Additional file 2: Table S1 — Sensitivity analysis. [file 1472-6947-14-47-S2.docx]

**Appendix 2**

| **Table 1. Estimates for γ** |
| --- |

|  | **Risk** | **Mean** | **Median** | **Min** | **Max** |
| --- | --- | --- | --- | --- | --- |
| **PE** | *High* | 0.29 | 0.25 | 0 | 1 |
|  | *Low* | 0.55 | 0.60 | 0 | 1 |
|  | *Baseline* | 0.50 | 0.45 | 0.09 | 1 |
| **AML** | *High* | 0.33 | 0.23 | 0 | 1 |
|  | *Low* | 0.54 | 0.46 | 0.01 | 1 |
|  | *Baseline* | 0.49 | 0.33 | 0.09 | 1 |

**Results (additional information)**

| **Table 2. Sensitivity analysis** | | | | | | | | | | | | | | | |
| --- | --- | --- | --- | --- | --- | --- | --- | --- | --- | --- | --- | --- | --- | --- | --- |
|  |  | **Pulmonary Embolism** | | | | | | |  | **Acute Myeloid Leukemia** | | | | | |
| **Group** | **Case** | **Treat (%)** | | **No treat (%)** | | | | **p-value** |  | **Treat (%)** | | **No treat (%)** | | | **p-value** |
| **By years of experience** | |  |  |  |  |  | |  |  |  |  |  |  |  |  |
|  | **>10 years of experience (N=21)** | | | | |  | |  |  |  |  |  |  |  |  |
|  | Base case | 21 | (100) |  | 0 | (0) | |  |  | 13 | (62) |  | 8 | (38) |  |
|  | High risk case | 8 | (38) |  | 13 | (62) | | <0.001 |  | 5 | (24) |  | 16 | (76) | 0.057 |
|  | Low risk case | 21 | (100) |  | 0 | (0) | | 1 |  | 18 | (86) |  | 3 | (14) | 0.125 |
|  | **<10 years of experience (N=20)** | | | | |  | |  |  |  |  |  |  |  |  |
|  | Base case | 19 | (95) |  | 1 | (5) | |  |  | 14 | (70) |  | 6 | (30) |  |
|  | High risk case | 8 | (40) |  | 12 | (60) | | <0.001 |  | 3 | (15) |  | 17 | (85) | 0.003 |
|  | Low risk case | 20 | (100) |  | 0 | (0) | | 1 |  | 18 | (90) |  | 2 | (10) | 0.125 |
| **By specialization** | |  |  |  |  |  | |  |  |  |  |  |  |  |  |
|  | **Hematology/oncology (N=14)** | | | |  |  | |  |  |  |  |  |  |  |  |
|  | Base case | 13 | (93) |  | 1 | (7) | |  |  | 11 | (79) |  | 3 | (21) |  |
|  | High risk case | 8 | (57) |  | 6 | (43) | | 0.063 |  | 4 | (29) |  | 10 | (71) | 0.039 |
|  | Low risk case | 14 | (100) |  | 0 | (0) | | 1 |  | 12 | (86) |  | 2 | (14) | 1 |
|  | **Other (N=27)** |  |  |  |  |  | |  |  |  |  |  |  |  |  |
|  | Base case | 27 | (100) |  | 0 | (0) | |  |  | 16 | (59) |  | 11 | (41) |  |
|  | High risk case | 8 | (30) |  | 19 | (70) | | <0.001 |  | 4 | (15) |  | 23 | (85) | 0.008 |
|  | Low risk case | 27 | (100) |  | 0 | (0) | | 1 |  | 24 | (89) |  | 3 | (11) | 0.008 |
| **By experience with similar PE case** | | | |  |  |  | |  | **By experience with similar AML case** | | | | | | |
|  | **Experienced (N=30)** | |  |  |  |  | |  |  | **Experienced (N=14)** | | | | |  |
|  | Base case | 29 | (97) |  | 1 | (3) | |  |  | 11 | (79) |  | 3 | (21) |  |
|  | High risk case | 12 | (40) |  | 18 | (60) | | <0.001 |  | 4 | (29) |  | 10 | (71) | 0.039 |
|  | Low risk case | 30 | (100) |  | 0 | (0) | | 1 |  | 11 | (79) |  | 3 | (21) | 1 |
|  | **Did not experience (N=11)** | | |  |  |  | |  |  | **Did not experience (N=27)** | | | | | |
|  | Base case | 11 | (100) |  | 0 | (0) | |  |  | 16 | (59) |  | 11 | (41) |  |
|  | High risk case | 4 | (36) |  | 7 | (64) | | 0.008 |  | 4 | (15) |  | 23 | (85) | 0.008 |
|  | Low risk case | 11 | (100) |  | 0 | (0) | | 1 |  | 25 | (93) |  | 2 | (7) | 0.004 |
| **By experience with decision analysis** | | | |  |  |  | |  |  |  |  |  |  |  |  |
|  | **Experienced (N=29)** | |  |  |  |  | |  |  |  |  |  |  |  |  |
|  | Base case | 29 | (100) |  | 0 | (0) | |  |  | 22 | (76) |  | 7 | (24) |  |
|  | High risk case | 13 | (45) |  | 16 | (55) | | <0.001 |  | 6 | (21) |  | 23 | (79) | <0.001 |
|  | Low risk case | 29 | (100) |  | 0 | (0) | | 1 |  | 24 | (83) |  | 5 | (17) | 0.625 |
|  | **Not experienced (N=12)** | | |  |  |  | |  |  |  |  |  |  |  |  |
|  | Base case | 11 | (92) |  | 1 | (8) | |  |  | 5 | (42) |  | 7 | (58) |  |
|  | High risk case | 3 | (25) |  | 9 | (75) | | 0.008 |  | 2 | (17) |  | 10 | (83) | 0.453 |
|  | Low risk case | 12 | (100) |  | 0 | (0) | | 1 |  | 12 | (100) |  | 0 | (0) | 0.016 |
| **By disease randomization order** | | | |  |  |  | |  |  |  |  |  |  |  |  |
|  | **PE first (N=21)** |  |  |  |  |  | |  |  |  |  |  |  |  |  |
|  | Base case | 20 | (95) |  | 1 | (5) | |  |  | 11 | (52) |  | 10 | (48) |  |
|  | High risk case | 9 | (43) |  | 12 | (57) | | <0.001 |  | 3 | (14) |  | 18 | (86) | 0.039 |
|  | Low risk case | 21 | (100) |  | 0 | (0) | | 1 |  | 19 | (90) |  | 2 | (10) | 0.021 |
| **Table 2. Sensitivity analysis cont.** | | | | | | | | | | | | | | | |
|  |  | **Pulmonary Embolism** | | | | | | |  | **Acute Myeloid Leukemia** | | | | | |
| **Group** | **Case** | **Treat (%)** | | **No treat (%)** | | | | **p-value** |  | **Treat (%)** | | **No treat (%)** | | | **p-value** |
|  | **AML first (N=20)** | |  |  |  |  | |  |  |  |  |  |  |  |  |
|  | Base case | 20 | (100) |  | 0 | (0) | |  |  | 16 | (80) |  | 4 | (20) |  |
|  | High risk case | 7 | (35) |  | 13 | (65) | | <0.001 |  | 5 | (25) |  | 15 | (75) | 0.007 |
|  | Low risk case | 20 | (100) |  | 0 | (0) | | 1 |  | 17 | (85) |  | 3 | (15) | 1 |
| **By PE case order** | |  |  |  |  |  | |  | **By AML case order** | | | | | | |
|  | **High risk case first (N=21)** | | |  |  |  | |  |  | **High risk case first (N=21)** | | | | | |
|  | Base case | 20 | (95) |  | 1 | (5) | |  |  | 12 | (57) |  | 9 | (43) |  |
|  | High risk case | 9 | (43) |  | 12 | (57) | | <0.001 |  | 3 | (14) |  | 18 | (86) | 0.012 |
|  | Low risk case | 21 | (100) |  | 0 | (0) | | 1 |  | 19 | (90) |  | 2 | (10) | 0.016 |
|  | **Low risk case first (N=20)** | | |  |  |  | |  |  | **Low risk case first (N=20)** | | | | | |
|  | Base case | 20 | (100) |  | 0 | (0) | |  |  | 15 | (75) |  | 5 | (25) |  |
|  | High risk case | 7 | (35) |  | 13 | (65) | | <0.001 |  | 5 | (25) |  | 15 | (75) | 0.021 |
|  | Low risk case | 20 | (100) |  | 0 | (0) | | 1 |  | 17 | (85) |  | 3 | (15) | 0.625 |
| **By slider position** | |  |  |  |  |  | |  |  |  |  |  |  |  |  |
|  | **Slider set at 0% (N=19)** | | | | |  | |  |  |  |  |  |  |  |  |
|  | Base case | 18 | (95) |  | 1 | (5) | |  |  | 14 | (74) |  | 5 | (26) |  |
|  | High risk case | 9 | (47) |  | 10 | (53) | | 0.004 |  | 5 | (26) |  | 14 | (74) | 0.035 |
|  | Low risk case | 19 | (100) |  | 0 | (0) | | 1 |  | 18 | (95) |  | 1 | (5) | 0.125 |
|  | **Slider set at 100% (N=22)** | | | | |  | |  |  |  |  |  |  |  |  |
|  | Base case | 22 | (100) |  | 0 | (0) | |  |  | 13 | (59) |  | 9 | (41) |  |
|  | High risk case | 7 | (32) |  | 15 | (68) | | <0.001 |  | 3 | (14) |  | 19 | (86) | 0.006 |
|  | Low risk case | 22 | (100) |  | 0 | (0) | | 1 |  | 18 | (82) |  | 4 | (18) | 0.125 |
| **By meeting eligibility criteria** | | | | | | | | | | | | |  |  |  |
|  | **Met all inclusion criteria (N=39)** | | | | |  |  | |  |  |  |  |  |  |  |
|  | Base case | 38 | (97) |  | 1 | (3) |  | |  | 27 | (69) |  | 12 | (31) |  |
|  | High risk case | 16 | (41) |  | 23 | (59) | <0.001 | |  | 8 | (21) |  | 31 | (79) | <0.001 |
|  | Low risk case | 39 | (100) |  | 0 | (0) | 1 | |  | 35 | (90) |  | 4 | (10) | 0.021 |
|  | **Did not meet all inclusion criteria (N=2)** | | | | | |  | |  |  |  |  |  |  |  |
|  | Base case | 2 | (100) |  | 0 | (0) |  | |  | 0 | (0) |  | 2 | (100) |  |
|  | High risk case | 0 | (0) |  | 2 | (100) | 0.50 | |  | 0 | (0) |  | 2 | (100) | 1 |
|  | Low risk case | 2 | (100) |  | 0 | (0) | 1 | |  | 1 | (50) |  | 1 | (50) | 1 |
| **By answered questions logically** | | | | | | | | | | |  |  |  |  |  |
|  | **Answered questions logically (N=37)** | | | | |  |  | |  |  |  |  |  |  |  |
|  | Base case | 36 | (97) |  | 1 | (3) |  | |  | 25 | (68) |  | 12 | (32) |  |
|  | High risk case | 16 | (43) |  | 21 | (57) | <0.001 | |  | 8 | (78) |  | 29 | (22) | <0.001 |
|  | Low risk case | 37 | (100) |  | 0 | (0) | 1 | |  | 32 | (86) |  | 5 | (14) | 0.039 |
|  | **Answered questions illogically (N=4)*** | | | | | |  | |  |  |  |  |  |  |  |
|  | Base case | 4 | (100) |  | 0 | (0) |  | |  | 2 | (50) |  | 2 | (50) |  |
|  | High risk case | 0 | (0) |  | 4 | (100) | 0.125 | |  | 0 | (0) |  | 4 | (100) | 0.5 |
|  | Low risk case | 4 | (100) |  | 0 | (0) | 1 | |  | 4 | (100) |  | 0 | (0) | 0.5 |

**Additional information for manuscript Table 3**

The results seem to indicate that the fit of the EUT model is considerably poorer than that of the other two models. For example, the decision to treat the patient with PE or AML in high-risk (“high threshold”) case agrees with the personal EUT thresholds only in 16 (39%) and 8 (20%) of physicians, respectively. The percentage of agreement according to regret and dual processing threshold model was statistically significantly higher. For example, in the same scenarios (PE or AML, high-risk case) decision to treat agree with 76% and 61% of the physicians’ regret threshold. The corresponding numbers for dual threshold model is 73% and 98%, respectively. As it can be seen in Table 3, the decisions to treat is significantly less in agreement with the EUT threshold than with regret or dual threshold model both in case of PE and AML case scenarios. We also detected significantly more agreement with dual processing model in the case of AML, while no difference in the percentage of agreement was seen between regret and dual threshold model in the case of PE (See also Fig 2).
